# Supplementary material for: CPI203, a BET inhibitor, down-regulates a consistent set of DNA synthesis genes across a wide array of glioblastoma lines
Source: PLoS One. 2025 May 16;20(5):e0306846. doi: 10.1371/journal.pone.0306846 (PMC12083822; doi:10.1371/journal.pone.0306846)
Supplement: Supplementary Figure 1 — Delta AUC = AUC Day 3-AUC Day 28. The average delta AUC values for each compound were ranked and are shown. B. This study uses a panel of 14 glioblastoma/glioma lines that have been partially characterized. (PDF) [file pone.0306846.s001.pdf]

Supplementary Figure 1

A. Drug Screen

Delta AUC	Drug	Target
95	CPI 203	BET
93	OTX 015	BET
92	(+)-JQ1	BET
91	ML 228	HIF1alpha
85	Ciclopirox	Histone demethylase
82	I-BET 151	BET
80	Givinostat	HDAC
78	Decitabine	DNMT
71	Hesperadin	Aurora Kinase B
68	GSK 591	PRMT5
63	LMK 235	HDAC
57	LBH	HDAC
57	ZM 447439	Aurora Kinase B
55	AZD 1480	Jak2
51	Triptolide	RNAPII
50	Alexidine	PTPMT1
41	BAY 299	BET
37	SAHA	HDAC
37	Furamidine	PRMT1
35	A 485	p300
34	Bromosporine	BET
34	AZ 20	ATR kinase
32	Rucaparib	PARP
29	EPZ 015666	PRMT5
28	MS 275	HDAC
26	PFI 1	BET
25	GSK 2830371	WIP
24	JIB 04	Jumanji (JmJ)
21	PF 06726304	EZH2
16	CX08005	PTP1B
15	SirReal 2	SIRT
14	AG-120	IDH
13	Sephin 1	PPP1R15A
13	(-)-JQ1	Control

Delta AUC	Drug	Target
11	TP 064	PRMT4
10	CP 690550	JAK
10	S 2101	LSD1
9	MS 023	PRMT
9	PFI 3	SMARCA
9	AZD 2461	PARP
9	IDG-C35	IDH
8	UNC 1999	EZH2
8	I-BRD9	BET
8	EPZ 004777	DOT1L
7	GN 44028	HIF-1alpha
7	OG-L002	LSD1
6	IOX 2	HIF-1alpha
6	UNC 0646	GLP
6	TC JL 37	TYK2
6	BAZ2-ICR	BAZ2
5	5-Azacytidine	DNMT
5	Temozolomide	DNA methyltransferase
5	Mirin	MRN-ATM
5	VH 298	VHL
5	EML 425	p300
5	WDR5 0103	WDR5
5	NVS-CECR2-1	CECR2
5	LY 303511	Control
5	OICR 9429	WDR5
5	UNC 2400	Control
4	cis VH 298	VH 298
4	H 89	PKA
4	PCI 34051	HDAC
3	BAY 598	SMYD2
2	BIX 01294	GLP
2	P 22077	USP7
2	GSK 6853	BRPF1
2	MM 102	WDR5

Delta AUC	Drug	Target
2	GSK J1	JMJD3
1	PJ 34	PARP
1	JQEZ5	EZH2
1	KU 60019	ATM kinase
1	PFI 4	BRPF1
1	AG-881	IDH
1	Valproic acid,	HDAC
1	Ex-527	SIRT
0	GSK J2	GSKJ1
0	Sodium 4-Phenylbutyrate	HDAC
0	Tranylcypromine	LSD1
0	AZ 5704	ATM Kinase
0	RG 108	DNA methyl-transferase
0	A 196	SUV420H1
-1	NI 57	BRPF
-1	Resveratrol	COX
-1	I-CBP 112	p300
-1	UNC 0642	G9a/GLP
-1	PRT 4165	BMI
-1	BI 9564	BET
-1	GSK J4	KDM
-1	UNC 0224	G9a/GLP
-1	L Moses	PCAF
-1	TCS PIM-1 1	Pim-1
-1	UNC 2327	PRMT3
-2	TC-E 5002	KDM
-2	TP 472	BET
-3	Ryuvidine	SETD8
-3	BRD 73954	HDAC
-3	BVT 948	Tyrosine Kinase
-4	GSK LSD 1	LSD
-4	CI 994	HDAC
-4	IOX 1	Histone Demethylase

Delta AUC	Drug	Target
-5	SB 747651A	MSK1
-5	TC-S 7009	HIF-2allpha
-6	RN 1	LSD1
-9	SD 1008	JAK/STAT
-11	OF 1	BRPF1B

B. Cell Lines

Cell Line	Age	Sex	Grade	Primary Recurrent	EGFR	IDH	PTEN	MGMT	TCGA Class
HK252		F	IV (GBM)	Recurrent	WT	Mut		Methylated	Proneural
HK281	65	F	IV (GBM)	Recurrent		WT			
HK357	58	M	IV (GBM)	Recurrent	WT	WT	Het	Unmethylated	Mesenchymal
HK372	33	M	IV(GBM)	Primary	Amp	WT	Het	Unmethylated	Mesenchymal
HK385	48	M	IV(GBM)	Primary	EGFRVIII	WT		Unmethylated	Proneural
HK408	55	F	IV(GBM)	Primary	Amp	WT			Proneural
HK412	49	M	IV(GBM)	Primary	Amp EGFRVIII	WT			Mesenchymal
PDX22			IV(GBM)			WT			
PDX59			IV(GBM)			WT			
TS600			IV(GBM)			WT			
TS1156			IV(GBM)			WT			
G83			IV(GBM)			WT			
MG119				Primary		Mut			
BT142	38	M	III(OA)	Primary		Mut			Proneural
