# Supplementary material for: CPI203, a BET inhibitor, down-regulates a consistent set of DNA synthesis genes across a wide array of glioblastoma lines
Source: PLoS One. 2025 May 16;20(5):e0306846. doi: 10.1371/journal.pone.0306846 (PMC12083822; doi:10.1371/journal.pone.0306846)
Supplement: Supplementary Figure 2 — Initial dose was 500nM however it was necessary to reduce the dose in some cases due to excessive cytotoxicity. (PDF) [file pone.0306846.s002.pdf]

Supplementary Figure 2

Cell Line	Drug	Dose	Cell Line	Drug	Dose	Cell Line	Drug	Dose	Cell Line	Drug	Dose	Cell Line	Drug	Dose	Cell Line	Drug	Dose
G83	Control	500nM	PDX22	Control	500nM	PDX59	Control	500nM	TS1156	Control	500nM	252	Control	500nM	357	Control	500nM
G83	PF06726304	500nM	PDX22	PF06726304	500nM	PDX59	PF06726304	500nM	TS1156	PF06726304	500nM	252	PF06726304	500nM	357	PF06726304	500nM
G83	Decitabine	500nM	PDX22	Decitabine	500nM	PDX59	Decitabine	500nM	TS1156	Decitabine	500nM	252	Decitabine	500nM	357	Decitabine	500nM
G83	A485	500nM	PDX22	A485	500nM	PDX59	A485	500nM	TS1156	A485	500nM	252	A485	500nM	357	A485	500nM
G83	Ciclopirox	500nM	PDX22	Ciclopirox	500nM	PDX59	Ciclopirox	500nM	TS1156	Ciclopirox	500nM	252	Ciclopirox	500nM	357	Ciclopirox	500nM
G83	ML228	500nM	PDX22	ML228	500nM	PDX59	ML228	500nM	TS1156	ML228	500nM	252	ML228	500nM	357	ML228	250nM
G83	CPI203	500nM	PDX22	CPI203	250nM	PDX59	CPI203	500nM	TS1156	CPI203	500nM	252	CPI203	500nM	357	CPI203	500nM
G83	SirReal2	500nM	PDX22	SirReal2	500nM	PDX59	SirReal2	500nM	TS1156	SirReal2	500nM	252	SirReal2	500nM	357	SirReal2	500nM
G83	Givinostat	500nM	PDX22	Givinostat	500nM	PDX59	Givinostat	500nM	TS1156	Givinostat	250nM	252	Givinostat	500nM	357	Givinostat	500nM
G83	Rucaporib	500nM	PDX22	Rucaporib	500nM	PDX59	Rucaporib	500nM	TS1156	Rucaporib	500nM	252	Rucaporib	500nM	357	Rucaporib	500nM
G83	GSK591	500nM	PDX22	GSK591	500nM	PDX59	GSK591	500nM	TS1156	GSK591	500nM	252	GSK591	500nM	357	GSK591	500nM
G83	Alexidine	250nM	PDX22	Alexidine	500nM	PDX59	Alexidine	250nM	TS1156	Alexidine	250nM	252	Alexidine	250nM	357	Alexidine	250nM
G83	Hesperidin	500nM	PDX22	Hesperidin	250nM	PDX59	Hesperidin	250nM	TS1156	Hesperidin	250nM	252	Hesperidin	250nM	357	Hesperidin	250nM
HK281	Control	500nM	PDX43	Control	500nM	TS600	Control	500nM	BT142	Control	500nM	MGG-119	Control	500nM	385	Control	500nM
HK281	PF06726304	500nM	PDX43	PF06726304	500nM	TS600	PF06726304	500nM	BT142	PF06726304	500nM	MGG-119	PF06726304	500nM	385	PF06726304	500nM
HK281	Decitabine	250nM	PDX43	Decitabine	500nM	TS600	Decitabine	500nM	BT142	Decitabine	500nM	MGG-119	Decitabine	500nM	385	Decitabine	500nM
HK281	A485	500nM	PDX43	A485	500nM	TS600	A485	500nM	BT142	A485	500nM	MGG-119	A485	500nM	385	A485	500nM
HK281	Ciclopirox	500nM	PDX43	Ciclopirox	500nM	TS600	Ciclopirox	500nM	BT142	Ciclopirox	500nM	MGG-119	Ciclopirox	500nM	385	Ciclopirox	500nM
HK281	ML228	500nM	PDX43	ML228	500nM	TS600	ML228	500nM	BT142	ML228	250nM	MGG-119	ML228	250nM	385	ML228	250nM
HK281	CPI203	250nM	PDX43	CPI203	500nM	TS600	CPI203	500nM	BT142	CPI203	500nM	MGG-119	CPI203	500nM	385	CPI203	500nM
HK281	SirReal2	500nM	PDX43	SirReal2	500nM	TS600	SirReal2	500nM	BT142	SirReal2	500nM	MGG-119	SirReal2	500nM	385	SirReal2	500nM
HK281	Givinostat	500nM	PDX43	Givinostat	500nM	TS600	Givinostat	500nM	BT142	Givinostat	250nM	MGG-119	Givinostat	500nM	385	Givinostat	500nM
HK281	Rucaporib	500nM	PDX43	Rucaporib	500nM	TS600	Rucaporib	500nM	BT142	Rucaporib	500nM	MGG-119	Rucaporib	500nM	385	Rucaporib	500nM
HK281	GSK591	500nM	PDX43	GSK591	500nM	TS600	GSK591	500nM	BT142	GSK591	500nM	MGG-119	GSK591	500nM	385	GSK591	500nM
HK281	Alexidine	250nM	PDX43	Alexidine	250nM	TS600	Alexidine	250nM	BT142	Alexidine	250nM	MGG-119	Alexidine	250nM	385	Alexidine	250nM
HK281	Hesperidin	250nM	PDX43	Hesperidin	250nM	TS600	Hesperidin	250nM	BT142	Hesperidin	250nM	MGG-119	Hesperidin	250nM	385	Hesperidin	250nM
