# Supplementary material for: CPI203, a BET inhibitor, down-regulates a consistent set of DNA synthesis genes across a wide array of glioblastoma lines
Source: PLoS One. 2025 May 16;20(5):e0306846. doi: 10.1371/journal.pone.0306846 (PMC12083822; doi:10.1371/journal.pone.0306846)

Supplementary Figure 3

A. MELK Module (223 genes)

RPAP3	DNM1L	EZH2	EXOSC8	ESPL1	INCENP	CENPN	SIPA1L1
DBF4	TPX2	CHCHD3	UTP20	DHX9	CPSF7	MCM7	INKA2
LARS2	TMEM230	MEGF9	NCAPH	DNAJB2	CHEK1	FAM111A	CALM1
CALCOCO1	RCOR1	FBXW4	ZSCAN18	LRCH1	NCAPD3	PLK1	HMGN2
POLA2	ORC6	KPNB1	KHDRBS1	CALCOCO2	CENPU	CDT1	DCAF12
MATR3	CLSPN	CDK5RAP3	CLTA	RSAD1	SLC25A4	UBXN6	SMC5
DEPDC1B	NUP50	NUP88	ZWINT	CDK5RAP2	CENPH	RFWD3	TOP1.00
NSUN2	CDC6	NCAPG	CIT	TEX10	NUP205	CDH2	KIFBP
TPR	AAAS	MTCH2	CDKN2C	NCBP1	TIAM1	NETO2	HMGN1
MPHOSPH9	MSH2	FOXM1	TUBA1B	CTSV	ATAD2	SHCBP1	FANCG
GINM1	CDC5L	RAD51AP1	CSE1L	HINT2	ZC3H18	ESCO2	DHFR
RC3H2	HNRNPH3	TIMELESS	EMC3	KIF11	PKNOX1	CTPS1	ARPC1A
MCM10	CDC7	CDCA3	SEPTIN6	CEP55	TONSL	DCP2	PRKDC
ASPM	ABHD4	CMAS	GPR108	KIF20B	RACGAP1	EXO1	
IDH3G	RBM23	MCM3	MCM8	EXOC6	SPC24	DNAJC22	
TRIP13	DHRS7	KIF20A	HNRNPR	PCDH10	USP1	POLR2A	
PRKACA	ZC3H14	MACROH2A1	HP1BP3	GAS2L3	FUBP1	COA4	
HMMR	VRK1	HES1	PPAT	TICRR	ZNF326	RTKN2	
GTSE1	PPP4R3A	CNPPD1	ATP6V1F	ANP32E	AGL	KPNA2	
MLH1	CHD8	PPP1R7	NDUFA10	DTL	PPP4R2	TM2D3	
UNG	PNN	ICMT	DNMT1	PARP1	KIF15	KNTC1	
SPAG5	GINS1	CENPF	CAPN7	DDX46	PBRM1	BRI3BP	
UBE2T	MYBL2	CTSD	PRKAB2	G3BP1	TASOR	AP3M1	
GNB1	FAM83D	CCND2	GRSF1	PNRC1	CDC25A	H2AX	
TOLLIP	POLA1	DYNC2I2	NASP	CDCA5	VCP	SRSF10	
NDC80	SUPT20H	CNTRL	AP3B1	MEPCE	MELK	FAM111B	
XPO1	CDIPT	YPEL5	RFC3	NCAPG2	SKA3	TRRAP	
TNPO1	ASF1B	IDE	CCNB1	SSRP1	PSMC3	XRCC6	
OXCT1	ISYNA1	HELLS	ITGA7	MTA2	CPSF2	BLM	
SRRT	CDK6	MASTL	TROAP	EML3	AMOTL1	HNRNPAB	

B. FBXO5 Module (39 genes)

TACC3	PAPOLA	NEK2	CDCA8	KIF2C	CCNB2	RRM1	CDCA2
DEPDC1	ARMCX3	SET	SMC2	NUF2	CIP2A	CKAP2L	KIF18B
SPDL1	FBXO5	HJURP	KIF23	CCNA2	SGO2	BUB1	WDHD1
NDC1	TTK	DLGAP5	CENPE	SKP2	HNRNPK	HNRNPA3	MZT1
PRR11	CDC20	KNSTRN	DIAPH3	JMY	PCLAF	ZWILCH	

C. FBXO5 module has no overlap with LINCS knock-down/over-expression data

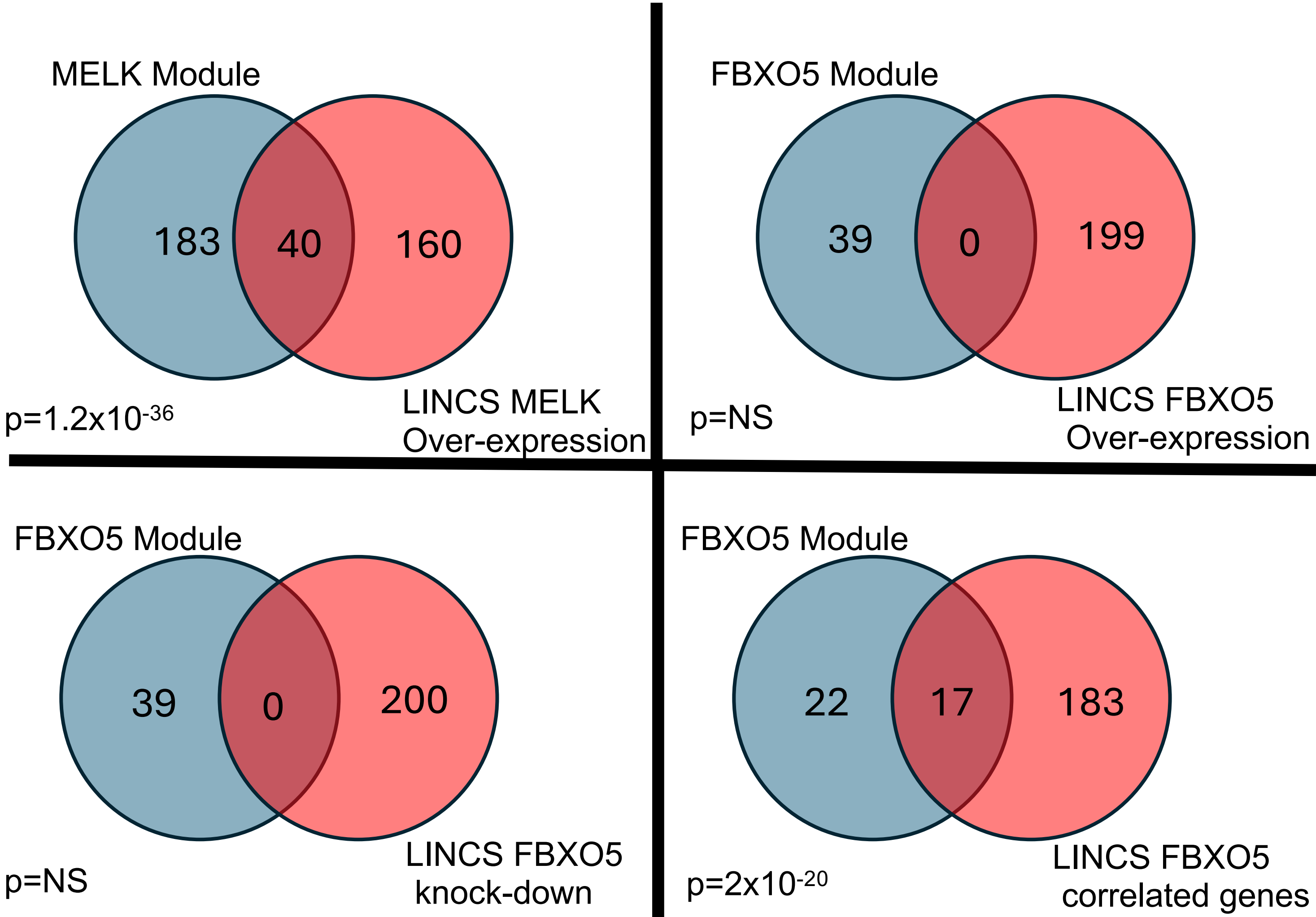

Supplement: Supplementary Figure 3 — B. The 39 genes identified in the FBXO5 module of the WCGNA analysis are listed. C. Overlap analysis shows a highly significant overlap between the MELK module and genes identified in MELK over-expression experiments (lincsproject.org). Overlap analysis between FBXO5 module and genes identified in FBXO5 over-expression or knock-down experiments shows no overlap. There was significant overlap between FBXO5 module genes and genes that are closely correlated with FBXO5 in publicly available expression data. (PDF) [file pone.0306846.s003.pdf]
