# Supplementary material for: CPI203, a BET inhibitor, down-regulates a consistent set of DNA synthesis genes across a wide array of glioblastoma lines
Source: PLoS One. 2025 May 16;20(5):e0306846. doi: 10.1371/journal.pone.0306846 (PMC12083822; doi:10.1371/journal.pone.0306846)
Supplement: Attachment — Submitted filename: PONE-D Review.docx [file pone.0306846.s004.docx]

**Reviewer Report: CPI203, a BET inhibitor, down-regulates a consistent set of DNA synthesis gene across a wide array of glioblastoma lines**

**Summary**

Glioblastoma is a complex disease that exhibits heterogeneity between patients and between tumors. To date, the standard of care treatment for glioblastoma is temozolomide. The varying response of temozolomide shows the need to develop new therapeutic targets and their associated compounds for glioblastoma treatment. Glioblastoma is propagated by the expression of pro-tumor growth factors (ie. EGFR) or deletion of tumor suppression genes (ie. PTEN, p53, Rb), the authors’ novelty utilizes chromatin modifying agents to identify new treatment options in glioblastoma. To identify viable chromatin modifying/epigenetic compounds as treatment options for glioblastoma, the authors screened 106 chromatin modifying/epigenetic compounds targeting 36 different genes first in independently derived gliomaspheres, lead compounds for each of the 12 compound class underwent a secondary screening using early passage of additional twelve gliomasphere lines. The authors calculated “delta AUC” to identify lead compound for each compound class. Of all compounds tested, CPI203, a BET class inhibitor reduced cell viability the most. RNA-seq of compound treated vs nontreated cells revealed that the same compound showed varying response in different cell line. However, CPI203, a BET class inhibitor shows the most consisted gene disruption in different cell lines. Further, the authors performed weighted correlation gene net analysis (WCGNA) ot identify sets of co-varying genes after compound treatment because genes often function through a network. WCGNA identified enriched genes “MELK” and “FBXO5” in sensitive cells, both of which as involved in mitotic and cell cycle, suggesting these two genes may serve as new therapeutic targets for glioblastoma treatment. Lastly, the author performed ATAC-seq in glioblastoma cell line after treatment with lead BET inhibitor(CPI203, JQ1, and OTX015). ATAC-seq showed a reduction of gene accessibility in cell lines after BET inhibitor treatment suggesting specificity of lead compounds.

**Comments**

1. In panel 1. The authors performed 106 compound screening in 5 cell lines. Each cell line was treated with 1uM or 10 uM for three days and 500 nM for twenty-eight days. Lead compound was identified by calculating “delta AUC” by the difference of day three and day twenty-eight by MTT assay.
   a. In the twenty-eight day treatment, were the cells passaged as they become confluent? If cells were passaged, then the conditions of the twenty-eight day treatment would be different compared to that of three day treatment. If the goal was to identify lead compound by three days in each cell lines, why not use a vehicle control such as DMSO or solvent used to dilute each compound, then compare cell viability at day three to the vehicle control.

**Minor Comments**

1. In figure 2F, the authors performed RNA-seq in compound treated vs control of each cell lines. PCA clustering analysis revealed two cluster of sensitive and resistant cells. Cluster 1, the sensitive cluster seems to have varying transcriptome across PCA1 whereas the cells in cluster 2 are more similar. Gene expression of cluster 2 may reveal additional gene sets in cluster 2 that can explain drug resistance.
   a. Cluster 1 was observed to express higher well-known tumor suppressor genes (CHKN2A/CDKN2B), could CPI203 serve as conditional therapy?
   b. Cluster 1 seems to have varied expression across PCA1. Could this suggest that CPI203 is binding to a specific target rather than a transcriptome similarity?
2. The authors made mentioned that the compounds used in the study were extensive but not exhaustive. Perhaps the authors should include rationale of why these compounds or specifically 106 was chosen.

**Other Comments**

Thank you for the opportunity for this review.
